# Supplementary material for: Digital transformation in schools of two southern regions of Sweden through implementation-informed approach: A mixed-methods study protocol
Source: PLoS One. 2023 Dec 19;18(12):e0296000. doi: 10.1371/journal.pone.0296000 (PMC10729980; doi:10.1371/journal.pone.0296000)
Supplement: Attachment — Submitted filename: Response to reviewers.docx [file pone.0296000.s001.docx]

Response to reviewers

Please include the following items when submitting your revised manuscript:

- A rebuttal letter that responds to each point raised by the academic editor and reviewer(s). You should upload this letter as a separate file labeled 'Response to Reviewers'.
- A marked-up copy of your manuscript that highlights changes made to the original version. You should upload this as a separate file labeled 'Revised Manuscript with Track Changes'.
- An unmarked version of your revised paper without tracked changes. You should upload this as a separate file labeled 'Manuscript'.

Dear Editor,

Thank you for the opportunity to revise the manuscript. We have attended to all comments raised by you and the reviewers. In this document we have included the comments raised by the editor and reviewers in normal text and responded to those in italics and blue directly under each point.

1. Please ensure that your manuscript meets PLOS ONE's style requirements, including those for file naming.

*We have changed the title, headings, figure legends and citations according to the style requirements of the Journal that the Editor has linked to.*

2. We note that the grant information you provided in the ‘Funding Information’ and ‘Financial Disclosure’ sections do not match.

When you resubmit, please ensure that you provide the correct grant numbers for the awards you received for your study in the ‘Funding Information’ section.

*We have corrected this.*

3. In your Data Availability statement, you have not specified where the minimal data set underlying the results described in your manuscript can be found. PLOS defines a study's minimal data set as the underlying data used to reach the conclusions drawn in the manuscript and any additional data required to replicate the reported study findings in their entirety. All PLOS journals require that the minimal data set be made fully available. For more information about our data policy, please see <http://journals.plos.org/plosone/s/data-availability>.

Upon re-submitting your revised manuscript, please upload your study’s minimal underlying data set as either Supporting Information files or to a stable, public repository and include the relevant URLs, DOIs, or accession numbers within your revised cover letter. For a list of acceptable repositories, please see [http://journals.plos.org/plosone/s/data-availability#loc-recommended-repositories](http://journals.plos.org/plosone/s/data-availability#loc-recommended-repositories). Any potentially identifying patient information must be fully anonymized.

Important: If there are ethical or legal restrictions to sharing your data publicly, please explain these restrictions in detail. Please see our guidelines for more information on what we consider unacceptable restrictions to publicly sharing data: [http://journals.plos.org/plosone/s/data-availability#loc-unacceptable-data-access-restrictions](http://journals.plos.org/plosone/s/data-availability#loc-unacceptable-data-access-restrictions). Note that it is not acceptable for the authors to be the sole named individuals responsible for ensuring data access.

We will update your Data Availability statement to reflect the information you provide in your cover letter.

*We have now created the repository and received a DOI number that we share here. We have not uploaded any documents yet and have not completed the repository with all the different instruments we are collecting. We have been in touch with our university responsible person for the repository to plan how to do this correctly as we have sensitive data that we cannot share, and we want to make sure that we do it without infringing on the GDPR. The DOI link is the following:* [*https://doi.org/10.5878/929p-cf12*](https://doi.org/10.5878/929p-cf12) *but since the DOI is not been assessed by the regulatory body yet, use this link instead to see the repository* [*https://snd.gu.se/sv/catalogue/dataset/preview/0b77896b-e5aa-40a2-b117-5896bd6d51f6/1*](https://snd.gu.se/sv/catalogue/dataset/preview/0b77896b-e5aa-40a2-b117-5896bd6d51f6/1) *The page is only available to you with the link. On page 30, we have simply added the name and link to the Swedish repository where the data are going to be available in the future.*

4. We note that the original protocol that you have uploaded as a Supporting Information file contains an institutional logo. As this logo is likely copyrighted, we ask that you please remove it from this file and upload an updated version upon resubmission.

*We are not sure which document the Editor is referring to. Usually the logos are permitted to be used without infringing on copyrights.*

Reviewers' comments:

Reviewer #1: Thank you for the opportunity to peer review this protocol. Some of my comments are suggested alternative ways of organising some of the work. These are only offered as suggestions that authors might consider, not as requirements for change.

*Thank you for your suggestions. We have taken into consideration all of the suggestions provided by the reviewer, and addressed most of those but also rejected some other suggestions. The explanations are presented below.*

Understandability
- I have read the protocol several times and it still feels quite unclear to me. Authors should attempt to make the intended activity easier to grasp for an external reader, perhaps through a visual flow chart of the activity that is planned and indicating which stakeholders are involved when and how. Because this is a large project over many years, it might be helpful to break down the work into smaller work packages, with specified input/output. Authors should consider creating a logic model, that ties the innovations, intervention and related activities more clearly to the “core components” (intermediary effects?) and desired outcomes.

*We have created a logic model, which resulted in a complex picture because of the many parts of the program. The logic model is now under review in another publication, so we have not included it in this submission in order to avoid publication/copyright issues. We are attaching a picture of the logic model here for information.*

- Practice profile: I don’t understand what this is.

*We have added a couple of sentences with an example in the section Practice Profile to hopefully clarify the concept of Practice Profile. Page 18.*

What is already known/prior research
This protocol lacks description of what we know from earlier research about interventions to improve uptake of digital technology. The questions I had after reading this several times were:
- Why should teachers be using DLM (other than the government having decided that)? There may be many good reasons, but these are not presented.

*We have added a few sentences on this on page 5 and 6.*

- Why should teachers be using LADs (other than this research proposal having been funded to implement these)?

*We have added two sentences on this on page 6.*

- What do we know about why this behaviour change in schools not been happening (is it lack of motivation, capability, opportunity?) What do we know about what teachers think (about the usefulness, barriers, facilitators, of using digital materials in teaching)?

*We have added a paragraph on barriers and facilitators on page 4.*

- Where did the idea of School Teams intervention come from? Has it been tried elsewhere in the context of research (if so, has it been proven to be effective?)

*We have added two sentences on the school team and its impact on page 15.*

- What do we know about other interventions to improve digital technology uptake in the classroom that could be alternatives to School Teams? (Is there a systematic review? Have authors reviewed the primary literature?)

*Yes, we have done that. We have only found one systematic scoping review by Albers and Pattuwage, which we cite as [16] in our reference list. Four other systematic reviews take up barriers and facilitators to the uptake of digital technology, which are crucial parts of the implementation process. However, those reviews do not use implementation frameworks. So, we did not present them in the manuscript.*

Quasi-experiment vs. randomisation
- In this study, schools would be self-selecting to receive the intervention, rather than randomized. This can have an effect on the results in the favour of the intervention. For instance, schools that are already motivated will be more likely to participate, and they would then be compared to schools that were not so motivated to use digital technology. Authors should acknowledge the weaknesses of a non-randomised design and describe what measures they will take to compensate. (e.g. consider before-after or time-series analysis of intervention and control group). Additionally, final results may be less likely to be applicable to schools that are not already motivated, and therefore do not sign up to participate – this should be discussed.

*This is a good point. We had already considered a before-after study of the experimental schools, but we did not explain that in the manuscript. We have now added a sentence about that on page 9 and discussed it on page 30.*

LAD co-design/development/evaluation
- To an outsider, it seems to me that DLM is a very broad term covering any learning material that is digital, while LAD is a specific product or solution that exists in a beta form (i.e. described “SAVis: a Learning Analytics Dashboard with Interactive Visualization and Machine Learning”). Is that correct? It needs clarifying.

*The reviewer is correct about DLMs. But also LADs are general products. Sometimes those are built together in one and the same system. Those are explained on page 5 and 6. The example that we have of SAVis (page 8) is just to demonstrate what we could do in terms of developing a LAD. However, we do not have a LAD for the project, and we intend to develop one during the pilot part of the project, as explained on page 20-21. We believe that the responses to previous comments have clarified this.*

- It is not clear to me why development of LAD would be done within a quasi-experiment design, and the LAD described in the article “SAVis…” appears to not be developed to the point of being suitable for use by people who are not digitally savvy (it looks like a tool for computer experts, not for teachers who are not using much digital technology). Before embarking on a multi-school experiment, my preference would have been to establish a preliminary “development” work package for LAD that included:
o Needs assessment regarding LAD
o Design development: Co-design with groups of key stakeholders (e.g. teachers, principles, school ICT administrators, maybe students and parents too?) involving several cycles of rapid iteration, user testing, and improvement of the LAD, to ensure that the solution is useable, useful, accessible, desirable, and suited/fit for use in schools’ teaching and learning environments.
o Piloting of LAD use by teachers and principles, with data collection (e.g. observation, interviews, logs)
o Assess the effect of LAD on teachers/school principles use, satisfaction, and on student outcomes if possible, by conducting a randomised trial , to understand if this innovation has the intended effect, before implementing it broadly. (You should be able to show that this LAD product has the desired effect on a set of outcomes, before setting about to implementing it on a broad scale).

*The reviewer might have missed the development of the LAD on page 20-21. We co-design the LAD in the same fashion as explained by the reviewer. We have modified the text page 8 and page 20-21 to hopefully clarify the development sequence.*

School team intervention – development and evaluation before implementation?
- It might be prudent to carry out a preliminary study as I have suggested above (co-development of the School Team intervention by a group of pilot schools, followed by assessment of effect using randomized trial methodology), before seeking to implement this intervention on a broad scale in a non-randomised experiment. Perhaps authors have intended this, but I have not understood it correctly.

*Yes, the idea of the pilot schools to test the implementation steps is presented on page 8. We have also added a new sentence to stress this.*

Reviewer #2: This paper describes a protocol that will be implemented in two areas of Sweden to document their school's digital transformation. The planned process has been thoroughly described and the research questions are clear and should provide comprehensive findings. The potential number of participants is quite large and, as such, will require detailed procedures such as outlined here.

While the protocol is well-considered, there are a number of general comments that need to be made. Overall the literature cited is dated. For example, the research that refers to 'the existing DLMs' was published in 2012. This is quite a long timeline for something as dynamic as technology implementation.

*We understand the issue raised by the reviewer, however, the published article dated 2012, Siemens & Baker, is a seminal paper defining LA for the first time, and still the reference used broadly today. About the other references about DLM, we used both older and new ones to show that the evidence has not really advanced in this field, like for example references 2 and 3 on page 4.*

The protocol makes little reference to the technology infrastructure roll out. I know little about the Swedish school system and it may be already very well resourced in this area and this is already in place? 'Stage 2: Installation' makes the only reference to 'Acquire resources' but there seems little mention elsewhere of infrastructure and specialist technical staff. These would be things that are likely to have a huge impact on the progress of the digital transformation of a school. There is a statement made that 'the intervention program does not require major organizational changes' so perhaps infrastructure and appropriate staffing is already in place.

*Good catch! We have added a couple of sentences on this topic on page 10, under the section Schools.*

I wonder if any provision has been made for cross-school comparisons when each local municipality is responsible for the purchase of resources? Will all schools be equally well-resourced in this case and how might any variations affect the research results?

*Every school in Sweden has a DLM in school. Its high or low usage is mostly due to teacher competences and willingness not accessibility. This is where this project comes in. Since each municipality decides what DLM to buy, we have nothing to say about the purchase. As you rightly suggested, we will compare teachers attitudes to use of specific DLMs to help the DLM companies to further develop their product and help them develop more qualitative DLM. This will therefore be a result of the research, hopefully.*

I also note that the EdTech 'companies are full research partners and have a say on the running of the researach work'. This will have to be managed carefully to ensure there is no conflicts of interest.

*The reviewer is right. We have been careful and have taken great measure in drawing a clear and standard contract with the CEOs of the EdTech Companies and the head of the department to make sure that it is clear what is expected of each other. This is mentioned at the beginning of the Edtech companies section on page 11.*

This will be a significant project and the results will potentially have wide-ranging impact. I look forward to reading about it further in the future.

*We do too! Thanks.*

7. PLOS authors have the option to publish the peer review history of their article ([what does this mean?](https://journals.plos.org/plosone/s/editorial-and-peer-review-process#loc-peer-review-history)). If published, this will include your full peer review and any attached files.


If you choose “no”, your identity will remain anonymous but your review may still be made public.


**Do you want your identity to be public for this peer review?** For information about this choice, including consent withdrawal, please see our [Privacy Policy](https://www.plos.org/privacy-policy).

Reviewer #1: No

Reviewer #2: **Yes:** Leanne Cameron
